# Reprogramming rather than depleting immunity: Targeting the CD38–NAD^+^ axis in antibody‐mediated autoimmunity

**DOI:** 10.1002/ctm2.70808

**Published:** 2026-08-31

**Authors:** Huiyuan Li, Jun Wei, Lei Zhang

**Affiliations:** ^1^ State Key Laboratory of Experimental Hematology National Clinical Research Center for Blood Diseases Haihe Laboratory of Cell Ecosystem Tianjin Key Laboratory of Gene Therapy for Blood Diseases Institute of Hematology & Blood Diseases Hospital Chinese Academy of Medical Sciences & Peking Union Medical College Tianjin China; ^2^ Tianjin Institutes of Health Science Tianjin China

1

Autoimmune diseases are driven by aberrant immune responses, but effective immunity is also essential for host defence, immune memory and tissue homeostasis. Their treatment therefore faces a persistent dilemma: How to suppress pathogenic immunity without compromising protective immune function. Corticosteroids and conventional immunosuppressants control disease largely through broad immune inhibition, whereas B‐cell‐ and plasma‐cell‐directed therapies provide greater cellular selectivity but still deplete immune‐cell compartments that also support normal humoral immunity. ^1^ Repeated or prolonged B‐cell depletion is manifested with persistent hypogammaglobulinemia, recurrent infections, impaired vaccine responses and immunoglobulin replacement in some patients. ^2^, ^3^ The unmet need is therefore not simply for stronger immunosuppression but for strategies that selectively attenuate disease‐driving immune functions while preserving immune competence. Because immune‐cell pathogenicity reflects not only cellular identity but also plastic functional states, reprogramming these states may offer an alternative to cell depletion. Immunometabolic pathways are particularly attractive in this regard, as they directly shape immune‐cell activation and effector function. ^4^

Plasma‐cell depletion provided the original rationale for targeting CD38 in immune thrombocytopenia (ITP), with the expectation that eliminating antibody‐secreting cells would reduce pathogenic autoantibody production. However, both our investigator‐initiated study and the subsequent randomised controlled trial showed that anti‐CD38 treatment induced platelet recovery within days, beginning as early as day 3. ^5^, ^6^ Such rapid kinetics appeared difficult to explain solely by plasma‐cell depletion and reduced autoantibody production. This temporal pattern therefore raised a broader mechanistic question: Might CD38 regulate not only the source of pathogenic antibodies but also the downstream effector phase of platelet destruction? Building on this clinical observation, our recent study delineated a CD38–nicotinamide adenine dinucleotide (NAD⁺)–macrophage differentiation axis and tested oral nicotinamide mononucleotide (NMN) as a non‐depleting strategy to therapeutically modulate this axis in ITP (Figure 1).

**FIGURE 1:**
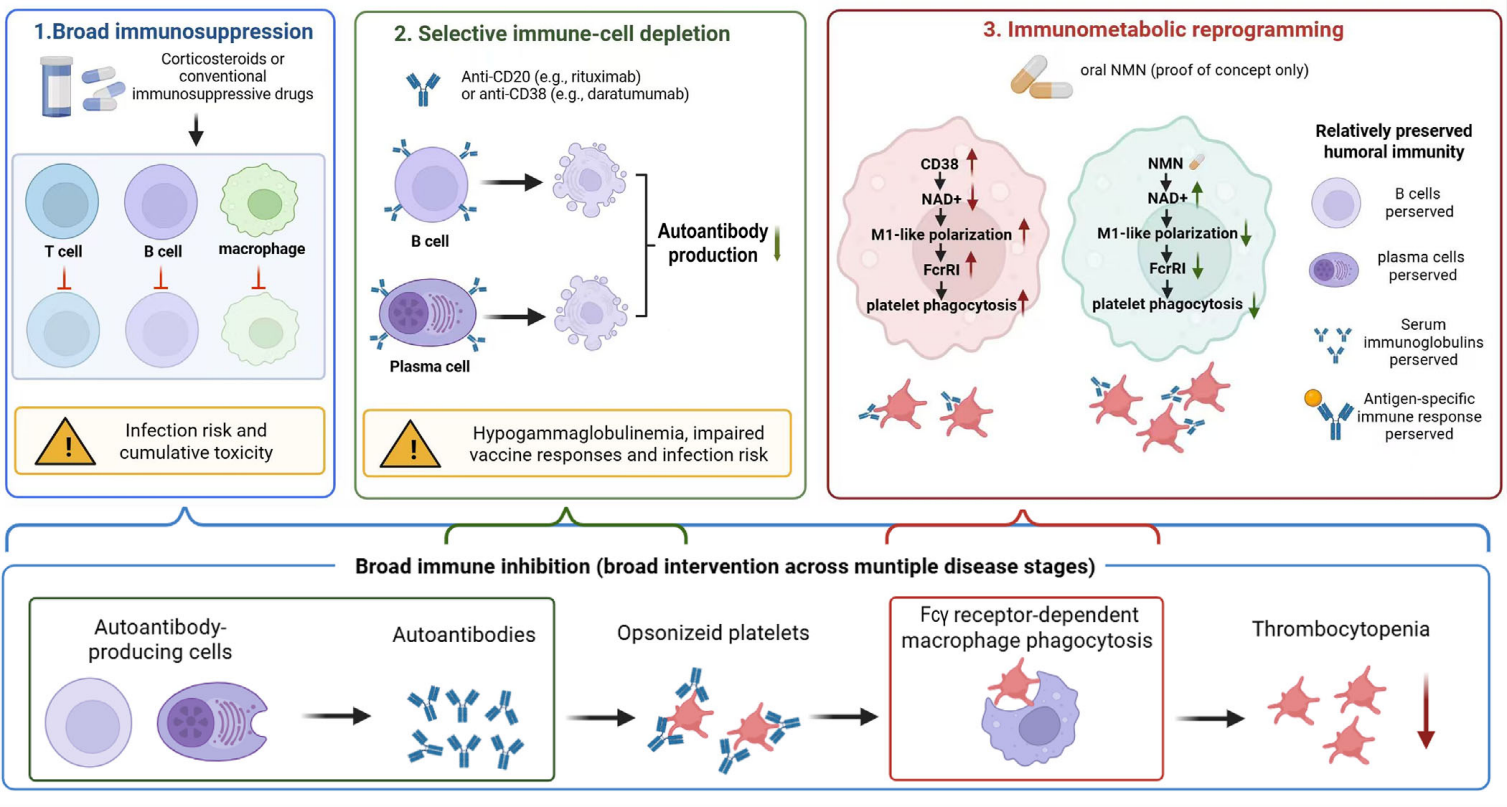
Therapeutic strategies for antibody‐mediated ITP: from broad immunosuppression and immune‐cell depletion to immunometabolic reprogramming. Conventional corticosteroids and immunosuppressive agents broadly inhibit multiple immune‐cell populations and may achieve disease control at the cost of impaired immune function, cumulative toxicity and increased susceptibility to infection. B‐cell‐ and plasma‐cell‐directed therapies, such as anti‐CD20 and anti‐CD38 antibodies, more selectively deplete immune‐cell populations involved in autoantibody production, but prolonged depletion can impair humoral immunity, contributing to hypogammaglobulinemia, impaired vaccine responses and infection risk. In contrast, immunometabolic reprogramming targets the downstream effector phase of platelet destruction without eliminating immune‐cell populations. By augmenting intracellular NAD^+^ availability, oral NMN attenuates CD38‐associated M1‐like macrophage polarisation, reduces FcγRI expression and limits Fcγ receptor‐dependent phagocytosis of opsonised platelets, thereby promoting platelet preservation without depleting macrophages. In the proof‐of‐concept setting, circulating B cells and plasmablasts, serum immunoglobulins and antigen‐specific humoral responses were relatively preserved. The lower panel summarises the shared pathogenic cascade of antibody‐mediated ITP, from autoantibody production and platelet opsonisation to Fcγ receptor‐dependent macrophage phagocytosis and thrombocytopenia, highlighting the distinct points of intervention of the three therapeutic strategies. Figure created using biorender.com.

In antibody‐mediated ITP, autoantibodies opsonise platelets and promote their clearance, whereas splenic macrophages act as key effectors by recognising and phagocytosing these opsonised platelets via activating Fcγ receptors, particularly FcγRI and FcγRIII.^7^ Beyond its established expression on plasma cells, CD38 is also a major NAD^+^ hydrolase whose upregulation in inflammatory macrophages drives NAD^+^ consumption and inflammatory programmes. ^8^ The study linked the CD38‐mediated NAD^+^ metabolism to macrophage‐mediated platelet clearance in ITP by showing a proinflammatory shift in splenic macrophages and demonstrating that genetic deletion or pharmacological inhibition of CD38 preserved intracellular NAD^+^ and reduced Fcγ receptor‐dependent clearance of opsonised platelets. NMN‐mediated NAD^+^ restoration recapitulated these effects by suppressing nuclear factor kappa B (NF‐κB)‐associated programmes, lowering FcγRI expression and limiting platelet phagocytosis without reducing macrophage abundance. Together, these findings position the CD38–NAD^+^ axis as a metabolic regulator of antibody‐mediated platelet destruction. More broadly, they suggest that macrophage pathogenicity reflects a reversible functional state that may be reprogrammed rather than eliminated.

These mechanistic findings raised a translational question: could restoring NAD^+^ reproduce the non‐depleting effects of CD38 inhibition without the broader effects of anti‐CD38 antibodies? NMN was therefore used to test whether NAD^+^ restoration could modify disease activity while preserving antibody‐producing cells and humoral immunity, thereby potentially reducing the risks of hypogammaglobulinemia and infection associated with plasma‐cell depletion. In the Phase 1/2 study, low‐dose oral NMN administered for 14 days was generally well tolerated in 25 patients with ITP, and 20% met the prespecified platelet‐response endpoint within 2 weeks, with additional patients showing early or sustained platelet improvement. These findings support biological activity and preliminary clinical proof of concept, but efficacy remains unconfirmed. Importantly, circulating plasmablasts and serum immunoglobulin levels remained stable. In the ovalbumin immunisation model, NMN did not reduce germinal‐centre B‐cell formation or ovalbumin‐specific immunoglobulin M (IgM) and immunoglobulin G (IgG) responses, providing evidence that short‐term NMN treatment did not measurably impair antigen‐specific humoral responses. Together, these findings suggest that pathological immune effector function may be attenuated without broad disruption of antibody‐mediated host defence.

The translational value of immunometabolic reprogramming will depend less on whether it can replace potent immunotherapies than on where it can complement them. The present study provides a potential framework for functionally selective treatment, in which a disease‐associated metabolic and inflammatory state is modulated while immune‐cell populations and protective immune responses are preserved. Potential future roles may include adjunctive or maintenance strategies to reduce exposure to broader immunosuppression, particularly when preservation of immune competence is a priority; however, these applications remain speculative and require prospective validation. ITP provides a tractable proof‐of‐concept setting because autoantibody‐mediated injury, macrophage‐dependent platelet clearance and treatment responses can be measured directly. However, the findings should not be extrapolated indiscriminately across autoimmune diseases. Initial evaluation may be most informative in antibody‐mediated disorders in which macrophages and Fcγ receptor‐dependent pathways play a major pathogenic role. Equally important, metabolic intervention should not be viewed as nonspecific supplementation. Its development as a precision strategy will require biomarkers of pathway‐dependence, potentially including CD38 activity, cellular NAD^+^ status, inflammatory macrophage programmes and FcγRI expression. Such stratification may ultimately define which patients are most likely to benefit from immunometabolic intervention.

Several limitations define the current evidence. The clinical study was small, single‐arm and open‐label, and spontaneous platelet variability or effects of stable background therapy cannot be fully excluded. The observed responses should therefore be interpreted as mechanistically informed proof of concept rather than efficacy confirmation. In addition, the regimen of 450 mg twice daily for 14 days was selected based primarily on prior human safety data rather than ITP‐specific dose optimisation; the appropriate dose, treatment duration, retreatment strategy and need for maintenance remain unknown. Heterogeneous responses also suggest that only a subset of patients may have therapeutically relevant CD38–NAD^+^ dysregulation. Future trials should establish pharmacodynamic target engagement and determine whether NAD^+^ metabolic or macrophage changes track with individual platelet responses. They should also evaluate whether baseline NAD^+^ status, CD38 activity, inflammatory macrophage programmes or FcγRI expression can identify patients most likely to benefit. Longer follow‐up and repeated‐dose studies should assess infections, immunoglobulin levels, haematological and metabolic parameters, durability of pharmacodynamic effects and interactions with concomitant therapies. Randomised dose‐finding trials incorporating longitudinal clinical, immune and metabolic monitoring will be required to define efficacy, optimise treatment exposure and assess long‐term safety. Future studies should move beyond asking whether NAD^+^ augmentation works, towards defining for whom, when and how immunometabolic reprogramming can provide durable disease control without compromising host defence.

## CONFLICT OF INTEREST STATEMENT

The authors declare no conflicts of interest.

## FUNDING INFORMATION

This work was supported by grants from the National Natural Science Foundation of China (82570173).
